# Supplementary material for: Quantitative High-Throughput Screening Methods Designed for Identification of Bacterial Biocontrol Strains with Antifungal Properties
Source: Microbiol Spectr. 2022 Mar 7;10(2):e01433-21. doi: 10.1128/spectrum.01433-21 (PMC9045326; doi:10.1128/spectrum.01433-21)
Supplement: SUPPLEMENTAL FILE 1 — Supplemental material. Download SPECTRUM01433-21_Supp_1_seq9.pdf, PDF file, 0.5 MB [file spectrum01433-21_supp_1_seq9.pdf]

Supplementary material

Table S1. The minimal inhibitory cell concentration (MICC) was determined for *B. subtilis* BCF001, *B. amyloliquefaciens* BCF007, *B. velezensis* BCF015 and *B. paralicheniformis* BCF009, as the lower number of cells (CFUs) that inhibit growth of *F. culmorum, F. graminearum* and *B. cinerea* by co-cultivation. The MICC against each fungal species was converted to an inhibition score by formula #1. For quantification of the culture supernatant inhibition potency, an inhibition score was calculated by formula #2 for each bacterial strain and for each experimental cell-inactivation method.

	BCF001	BCF007	BCF009	BCF015
MICC (CFU)				
F. culmorum	1903	<339	>3.89*10^6	<497
F. graminearum	57	60	>1.09*10^5	74
B. cinerea	67	150	>1.10*10^5	153
Co-cultivation inhibition score				
F. culmorum	3.8	4.5	0.8	4.4
F. graminearum	3.4	3.4	0.4	3.3
B. cinerea	3.3	3.0	0.4	3.0
Supernatant inhibition score (antibiotics)				
F. culmorum	2.63	2.81	0.62	2.06
F. graminearum	2.14	1.57	-0.43	1.81
B. cinerea	3.53	3.43	1.75	3.50
Supernatant inhibition score (filtration)				
F. culmorum	2.52	2.64	0.46	2.13
F. graminearum	2.04	1.22	-0.50	1.81
B. cinerea	3.55	3.42	1.32	3.53
Supernatant inhibition score (filtration and antibiotics)				
F. culmorum	2.47	2.56	0.26	1.81
F. graminearum	2.35	1.37	-0.40	1.72
B. cinerea	3.50	3.35	0.81	3.50

Table S2. The significance of the minimal inhibitory cell concentration (MICC) against each fungus was determined for *B. subtilis* BCF001, *B. amyloliquefaciens* BCF007, *B. velezensis* BCF015 and

B. paralicheniformis BCF009 by students t-test comparison between the strains. Statistically significant values ($p<0.05$) are indicated in red.

Compared strains	*F. culmorum*	*F. graminearum*	*B. cinerea*
BCF001/BCF007	0.00	0.45	0.10
BCF001/BCF009	$2*10^{-64}$	0.04	0.04
BCF001/BCF015	$2*10^{-66}$	0.32	0.15
BCF007/BCF009	$2*10^{-64}$	0.04	0.04
BCF007/BCF015	$1*10^{-62}$	0.32	0.48
BCF009/BCF015	$2*10^{-64}$	0.04	0.04

Table S3. Statistical significance of different *Bacillus* supernatant sterilization methods on fungal inhibition results. Statistical significance was calculated using student's t-test. Statistically significant values ($p<0.05$) are indicated in red.

Filtered supernatants with antibiotics compared to supernatants with antibiotics						
Supernatant volume (µl)		10	20	40	60	80
F. culmorum	BCF001	0.43	0.55	0.69	0.18	0.12
	BCF007	0.21	0.01	0.24	0.82	0.11
	BFC009	0.97	0.2	0.05	0.08	0.01
	BFC015	0.77	0.31	0.09	0.01	0.04
F. graminearum	BCF001	0.88	0.09	0.01	0	0.94
	BCF007	0.31	0.14	0.66	0.85	0.06
	BFC009	0.3	0.44	0.45	0.61	0.78
	BFC015	0.28	0.01	0.6	0.34	0.32
B. cinerea	BCF001	0.11	0.61	0.4	0.89	0.71
	BCF007	0.81	0.51	0.08	0.34	0.34
	BFC009	0.07	0.1	0.02	0.1	0.22
	BFC015	0.46	0.51	0.06	0.82	1
Filtered supernatants with antibiotics compared to filtered supernatants						
Supernatant volume (µl)		10	20	40	60	80
F. culmorum	BCF001	0.77	0.04	0.97	0.52	0.84
	BCF007	0.02	0	0.06	0.02	0.87
	BFC009	0.07	0.13	0.62	0.97	0.09

	BFC015	0.42	0	0.18	0.09	0.04
F. graminearum	BCF001	0.39	0.2	0.01	0.03	0.32
	BCF007	0.42	0.75	0.08	0.05	0.98
	BFC009	0.29	0.8	0.04	0.91	0.37
	BFC015	0.09	0.93	0.71	0.63	0.72
B. cinerea	BCF001	0.55	0.71	0.52	0.35	0.27
	BCF007	0.46	0.82	0	0.11	0
	BFC009	0.24	0.01	0.47	0.22	0.33
	BFC015	0.15	0.89	0.03	0.35	0
Filtered supernatants compared to supernatants with antibiotics						
Supernatant volume (µl)		10	20	40	60	80
F. culmorum	BCF001	0.38	0.29	0.7	0.04	0.16
	BCF007	0.31	0	0.23	0.01	0.41
	BFC009	0	0.71	0.07	0.07	0.13
	BFC015	0.58	0.61	0.47	0.69	0.27
F. graminearum	BCF001	0.42	0.82	0.16	0.81	0.37
	BCF007	0.19	0.12	0.18	0.03	0.07
	BFC009	0.19	0.54	0.14	0.42	0.24
	BFC015	0.2	0.19	0.53	0.81	0.16
B. cinerea	BCF001	0.2	0.32	0.35	0.5	0.44
	BCF007	0.03	0.47	0.04	0.36	0
	BFC009	0.29	0.47	0.06	0.13	0.56
	BFC015	0.32	0.7	0.06	0.19	0.01

Supplementary figures

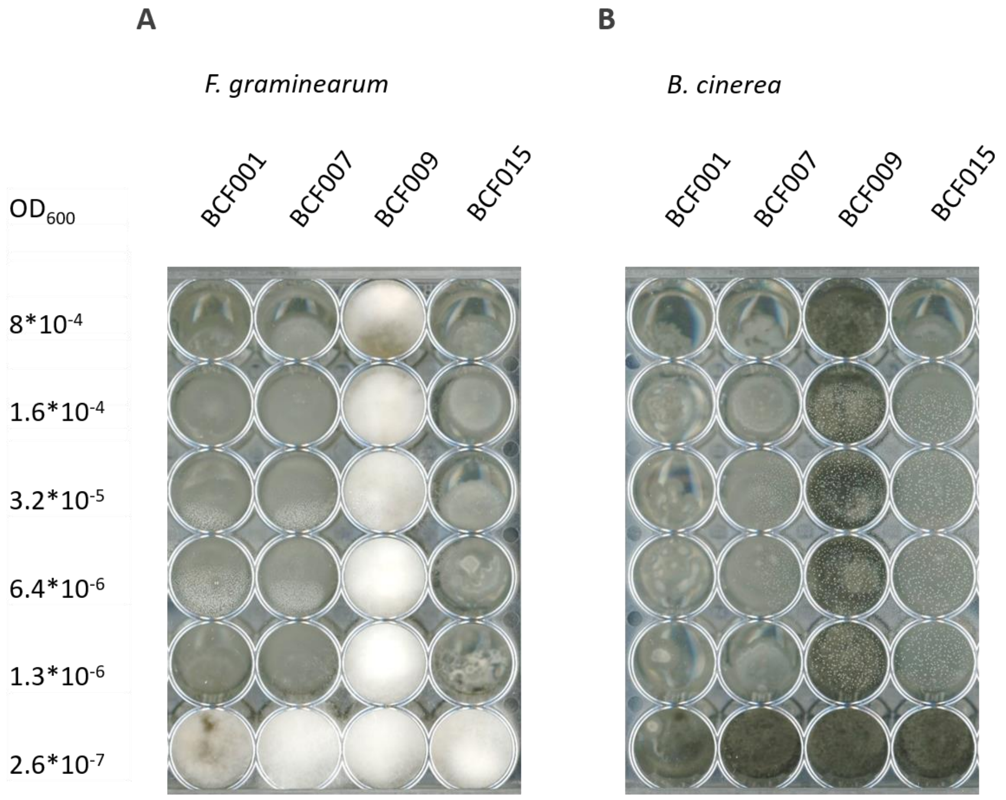

Fig. S1. Fungal inhibition assay with *Bacillus* dilution series. Dilution series of *B. subtilis* BCF001, *B. amyloliquefaciens* BCF007, *B. paralicheniformis* BCF009 and *B. velezensis* BCF015 were prepared and inoculated in consecutive columns of a 48-well microtiter plate. In each well, a constant spore concentration was inoculated of (A) *F. graminearum* and (B) *B.* cinerea. Pictures are representative of triplicates.

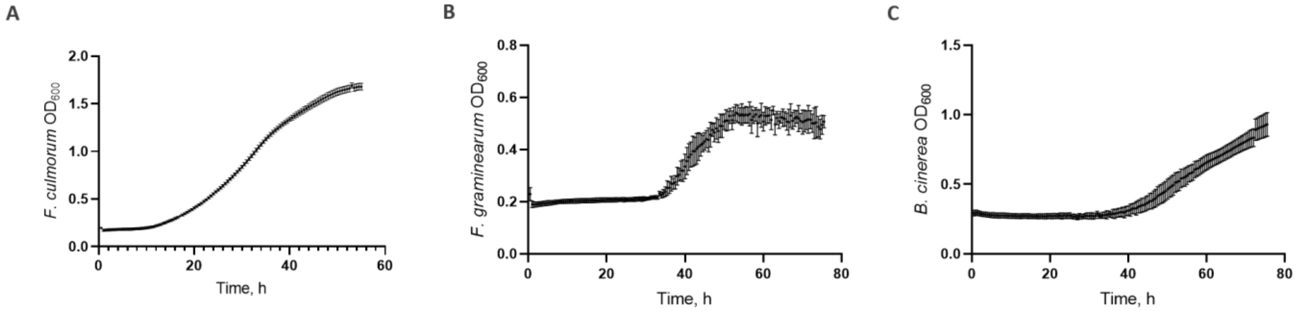

Fig. S2. Fungal growth monitored of A) *F. culmorum* B) *F. graminearum* and C) *B. cinerea* over 3 days by spectrophotometry using a 5x5 area scan protocol of each well. Subsequently the 25 measurements of each well were averaged.

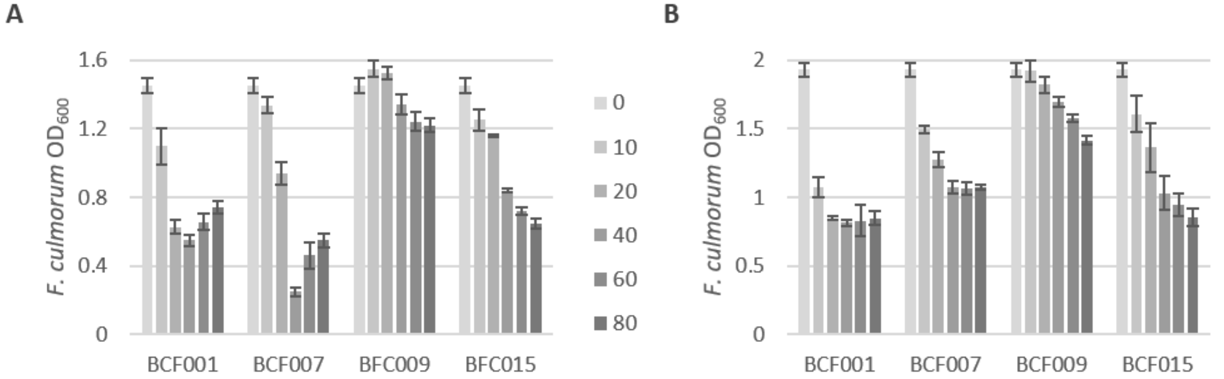

Fig. S3. Variation of *F. culmorum* growth with *Bacillus* supernatants. Inhibition assays with *F. culmorum* were prepared from different spore solutions and reached different final growth after 67h (A) and 72h (B) cultivation in PDB medium both with and without bacterial culture supernatants. Growth was evaluated by spectrophotometric measurements (OD_{600}). Standard deviations were calculated from biological triplicates.

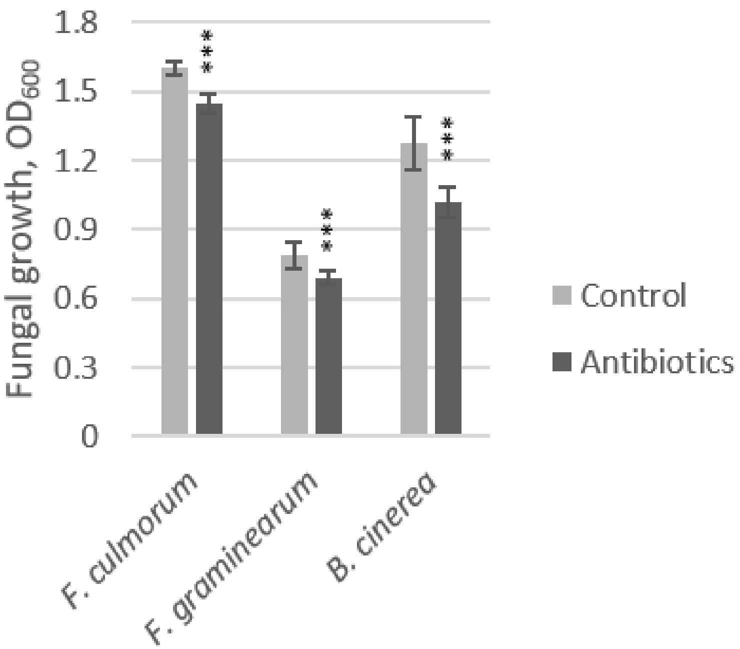

50 **Fig. S4.** Growth of *F. culmorum, F. graminearum* and *B. cinerea* without and without addition of

51 antibiotics (50 µg/ml chloramphenicol and 10 µg/ml tetracycline). ***P<0.0005

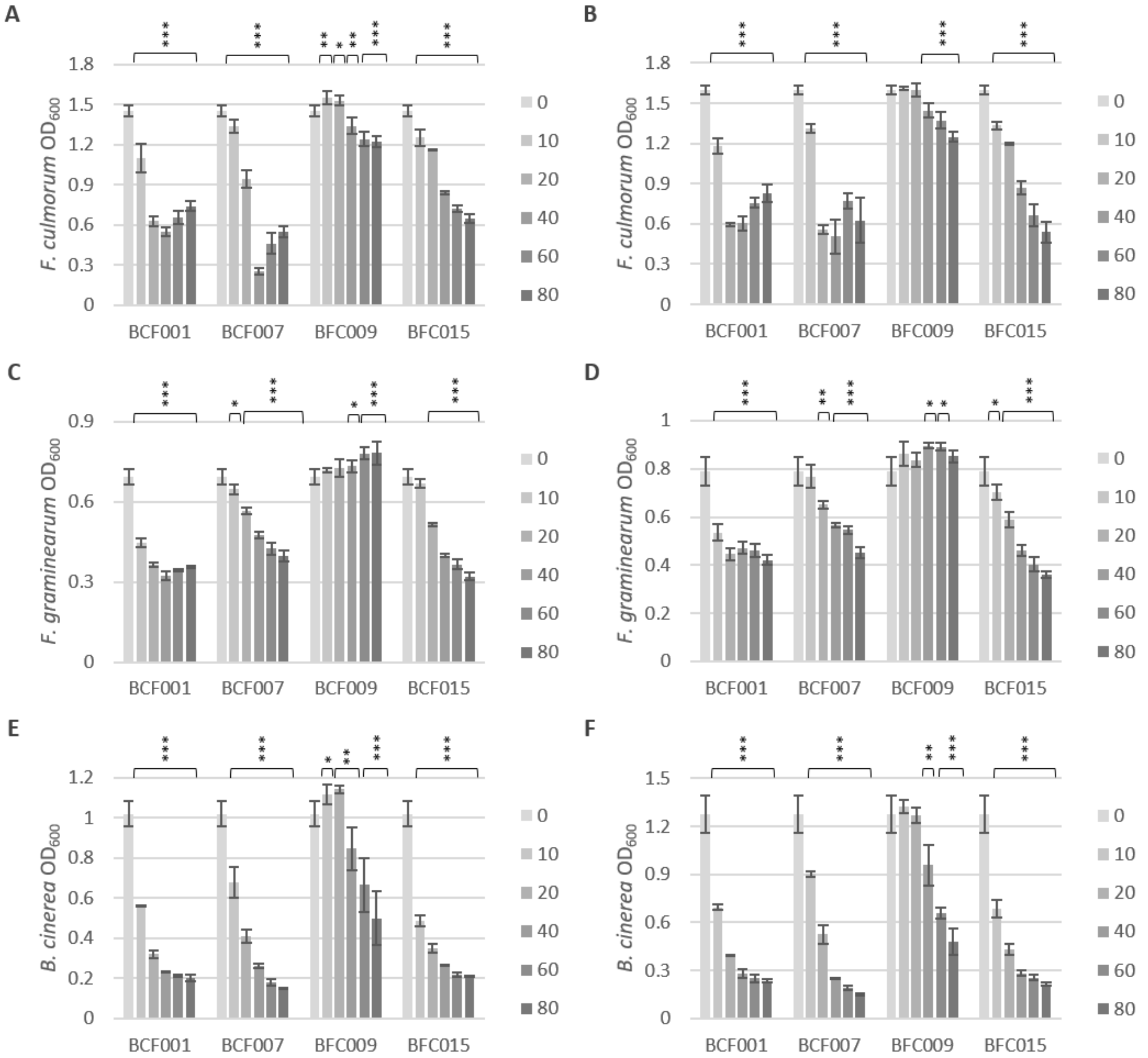

Fig. S5. Comparison of methods to circumvent bacterial cell growth in fungal inhibition assays with bacterial supernatants. Fungal spore suspensions of *F. culmorum* (A,B), *F. graminearum* (C,D) and *B. cinerea* (E,F) were inoculated with filtered bacterial culture with antibiotics (A, C, and E) or without addition of antibiotics (B, D, and F). *P<0.05, **P<0.005, ***P<0.0005.

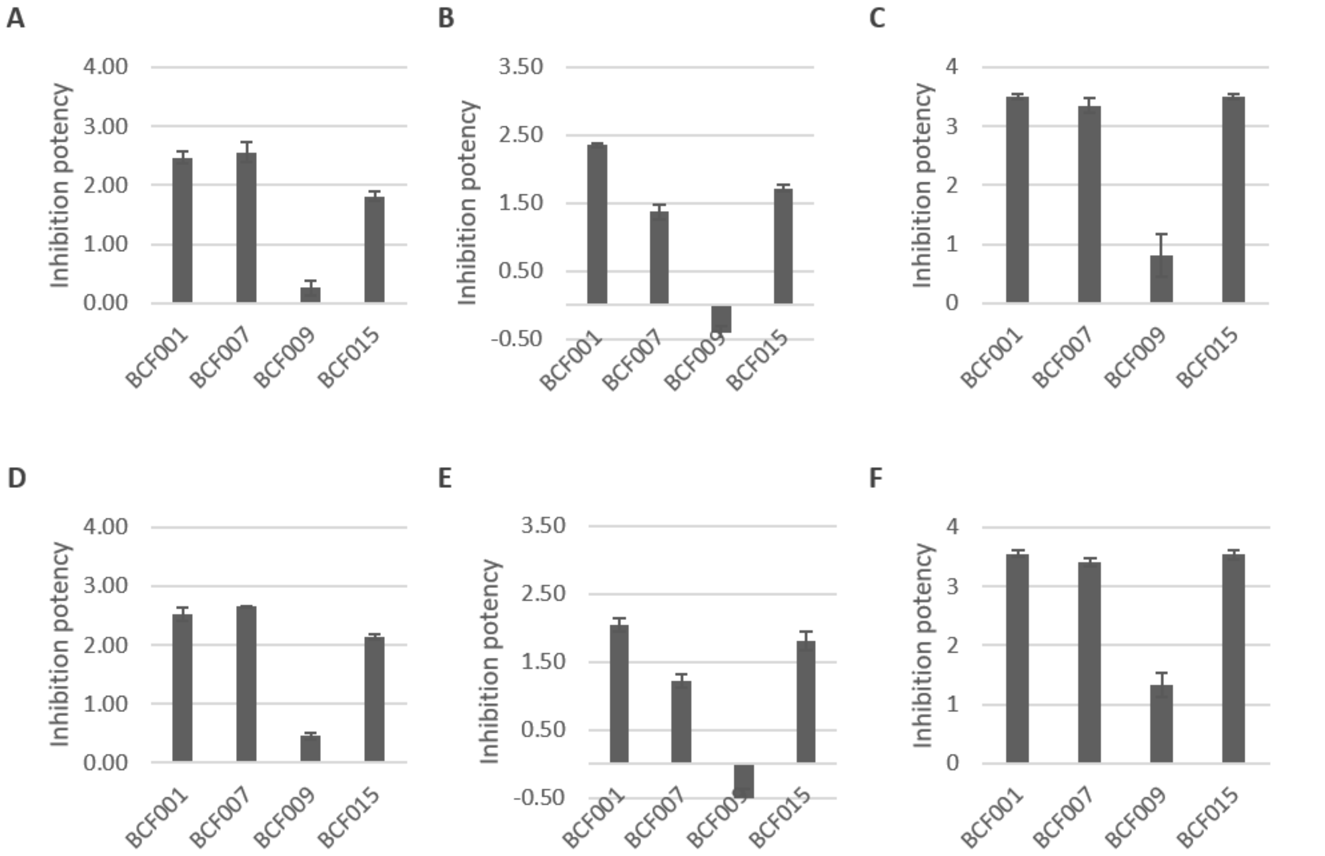

Fig. S6. Comparison of supernatant inhibition potencies using *Bacillus* cell inactivation by filtration and antibiotic addition or by filtration only. The culture supernatant inhibition potencies of *B. subtilis* BCF001, *B. amyloliquefaciens* BCF007, *B. paralicheniformis* BCF009 and *B. velezensis* BCF015 against *F. culmorum* (A,D), *F. graminearum* (B,E) and *B. cinerea* (C,F) were calculated by applying the formula #2. The effect of bacterial cell inactivation by filtration and antibiotic addition (A-C) was compared to bacterial cell inactivation by filtration (D-F).
